# Technology-based interventions on burden of older adults’ informal caregivers: a systematic review and meta-analysis of randomized controlled trials

**DOI:** 10.1186/s12877-024-05018-w

**Published:** 2024-05-04

**Authors:** Yang Zhou, Zhenggang Bai, Keyan Wan, Tianyi Qin, Rui He, Chengdan Xie

**Affiliations:** 1 Department of Sociology, School of Public Affairs, Nanjing University of Science and Technology, No. 200, Xiaolingwei District, Nanjing, 210094 Jiangsu Province China; 2 The Evidence-Based Research Center of Social Science & Health, Nanjing University of Science and Technology, No. 200, Xiaolingwei District, Nanjing, 210094 Jiangsu Province China

**Keywords:** Caregiver burden, Informal caregivers, Older adults, Technology-based interventions (TBI), Systematic review and meta-analysis

## Abstract

**Background:**

An increasing number of technologies are provided to reduce the burden of older adults’ informal caregivers. However, less is known about the effects and the mechanism of technology to work on burden. This review is to evaluate the effectiveness of technology-based interventions (TBI) in alleviating the burden of older adults’ informal caregivers and to distinguish its effective mechanism via group disparities.

**Methods:**

A systematic review and meta-analysis of randomized controlled trials studies (RCTs) has been conducted. Web of Science, PubMed, EMBASE, Scopus, CINAHL, PsycINFO, WANFANG, CNKI, CQVIP databases, Cochrane Library Trials, and ClinicalTrials.gov were searched for trial studies and registry in both English and Chinese published from January 1990 to October 2022. Reviewers independently screened the articles and trials, conducted quality assessments, and extracted the data. All processes were guided by Preferred Reporting Items for Systematic Reviews and Meta-Analyses guidelines. Risk of bias of the studies was evaluated by the Cochrane Systematic Review Handbook. The meta-analysis was conducted by RevMan 5.13. Subgroup analyses, sensitivity analyses, publication bias were also conducted.

**Results:**

A total of 11,095 RCTs were initially screened, and 14 trials representing 1010 informal caregivers were included finally. This review proved TBI effective in reducing caregiving burden older adults. Subgroup analysis showed effects of TBI differed by interventions on control group and medical conditions of care recipients.

**Conclusion:**

TBI is an effective way to alleviate the burden on informal caregivers of aging people. Interventions for control groups and medical conditions of care-recipients are significant factors in effective interventions. Future researches could include more trials with high-quality or to explore more targeted aging groups, modalities of TBI, or caregiver outcomes.

**Trial registration:**

The review protocol was registered on PROSPERO [CRD42021277865].

**Supplementary Information:**

The online version contains supplementary material available at 10.1186/s12877-024-05018-w.

## Background

Nowadays, the world’s increasing aging population implies growing needs for caregiving. Aging groups are usually diagnosed with medical conditions related to aging such as dementia, cancer, stroke, and chronic conditions [1]. A developing number of older people are leaving nursing home and being cared by families as a means of cutting down costs for caregiving. Family caregivers were regarded as an important extension of the aging healthcare system [2]. Informal caregivers, also known as family caregivers, are unpaid individuals (family members, friends) who provide most of the required assistance or supervision [2, 3]. Caring for older persons usually led to personal sacrifices, and caregivers produced goods to their loved ones and society at considerable costs to themselves [4]. It has been well documented that most caregivers undertook heavy burden, led to negative objective and subjective consequences resulting from the provision of care, encompassing physical, psychological, emotional, financial, and social problems [3]. Especially, during the COVID-19 pandemic, family caregivers experienced changed caregiving tasks and additional caregiving challenges within unusual circumstances and changes to the caregiving routines [5].

Globally, needs were urgent for enhanced capacity for caregivers of older adults and home care, which many believed could be addressed via technology at least in part. Gerontechnology as an expanding and novel field dedicated to the development and utilization of technological devices to meet the demands of aging groups [6]. Mahoney and colleagues [7] detected the effects of the first computerized workplace-based intervention for caregivers directly. In a study on an interview study of around 1500 caregivers, 53% of them used Internet sources of information upon caregiving [2]. A National Academy of Sciences report pointed out technology might be helpful for family caregivers in ways typical for the general population and specific functions for the caregiving role [8]. And previous studies were mostly focus on TBI used to support dementia caregiving [9–21] and by means of Internet [1, 9, 12, 13, 15, 16, 18, 19, 22–25]. Yet, there were not acknowledged definitions and clarifications on those applied technologies. And publications even used extensively variated terminologies [26]. For this research, we divided technologies for caregiving into several main formats: assistive devices (e.g., helping devices for transportations), tele-series devices (e.g., telephone etc.), Information and Communications Technologies (ICTs) (e.g., Internet etc.), smart home technology, and artificial intelligence and big data [21, 26]. Usage of technology applications were categorized as listed: for decreasing caregiving tasks and compensating for needy care-recipients; for delivering psycho-social interventions, mainly include psychosocial/cognitive behavioral therapy and social supports; for providing information and problem-solving strategies, coordination of care, and managing a new caregiving routine; for social and family communications and leisure; and for activities and behavioral training via devices [5, 8, 14, 18, 27].

TBI had the potentials of removing the barriers of traditional social services (e.g., logistics) and facilitating the utility of demanding supports for caregivers [2]. Findings recognized that TBI had the strengths of being convenient, practical, cost-effective, low-cost, and contained various formats and interactive between actors [13, 26, 28–30]. Technology has helped caregivers increase knowledge and skills, sense of control, confidence, care self-efficacy, and enjoyment; and improve their adherence to treatment, support from professionals and other caregivers; and connect more with distant family members [2, 29, 31, 32]. Overall, caregivers who received TBI reported a decrease in workload, pressure and burden in caregiving [2, 6, 30]. However, a substantial number of caregivers have experienced obstacles in using supportive technologies, such as limited accesses, availability of technology, cost, time, less willingness, problems of affordability, retrofitting complications, potential inappropriate use of the technology, and other usability problems [13, 27, 30, 33]. Participants indicated concerns about technology, like digital divide, considering of standardization, technicalities, surveillance, skepticism, and security [26, 27, 34]. Thus, it is essential to illustrate the roles of TBI to support informal caregivers of older adults generally.

Multiple systematic review and meta-analysis of technology interventions to support caregivers are increasingly noted in the literature [35]. While, most of reviews discussed the positive aspects of technologies on dementia patients and their family caregivers, and most of them focused on computer and Internet-based intervention [28, 36, 37]. Several reviews were about the effects of assistive technologies and mobile apps on caregivers [23, 38].

Accordingly, the effectiveness of TBI on the burden of caregivers of the elderly is inconclusive. Less is known about the effects of TBI on caregiving burden within aging care-recipients with various specific medical issues, and about the effects of diverse modalities of technologies. This review is to define the utilization of technologies applied to decrease caregiving burden, to detect their effects in practice, and to distinguish the influential elements of TBI on caregiving burden of aging people via several group disparities.

## Methods

This systematic review and meta-analysis comprehensively incorporated RCTs. The systematic review was registered with the International Prospective Register of Systematic Reviews at the Centre of Reviews and Dissemination in the UK (CRD42021277865). It was reported according to the 2020 edition of Preferred Reporting Items for Systematic Review and Meta-analyses (PRISMA) guidelines [39].

### Search strategy

The complete literature search was carried out on 11 databases and registry, containing Web of Science, PubMed, EMBASE, Scopus, CINAHL, PsycINFO, WANFANG, CNKI, CQVIP databases, Cochrane Library Trials, and ClinicalTrials.gov. We selected all articles and trials published between January, 1990 and October, 2022. Our search keywords consisted of three subsets: participant (“caregiving”, “caregiver”, “carer”), intervention (“tele”, “big data”, “mobile”, “internet”, “robot”, “technology”, “artificial Intelligence”, “e-health”, “m-health”, “e-medicine”, “m-medicine”), and outcomes (“burden”, “stress”). We connected them with two Boolean operators (AND and OR) to search for relevant researches in English and in Chinese.

### Study inclusion and exclusion criteria

Two teams of researchers screened and selected these studies independently. Studies were chosen in accordance with the inclusion criteria as follows: (1) Study designs were RCTs; (2) Informal caregivers of elderly people included family members, relatives, friends, and volunteers; (3) Interventions in trial groups were provided using supportive technology such as Internet, apps, network, and other technology products; (4) Articles were published between January, 1990, and October, 2022; (5) Interventions aimed at improving the burden of caregivers, measured by burden scales; (6) Care recipients were people aged 55 years or above. We excluded studies if they met the following criteria: (1) were published in neither Chinese nor English; (2) did not report results data, or could not be inferred by contents.

### Data extraction

All search results from databases were exported to EndNote X9.3.3 and Excel. We generated a data extraction table to collect the basic information of the included studies, containing title, author, country, year of publication, abstract, purpose of study, journal and so on. Also, the coding scheme extracted contents including subjects, intervention designs, outcomes, measurement tools and duration of follow-ups. We extracted the data mainly by quantitative data: mean and standard deviation (SD). Some of the literature did not report these data directly. The mean and SD of these studies were calculated according to the standard error (SE), D effect size, *P* value, 95% confidence interval (CIs) and other information given in the literature. Two dependent teams extracted the information separately. It was verified by another reviewer, to ensure the reliability of data extraction. All reviewers agreed on the content of the final data extraction table after discussion. The intricate particulars of data extraction were delineated in Additional file 1.

### Assessment of risk of bias

Risk of bias of each eligible study was further assessed by two independent reviewers following the *Cochrane Systematic Review Handbook*. This research quality assessment evaluated the levels of bias in the following: (1) selection bias (sequence generation and allocation concealment); (2) performance bias (blinding of participants and personnel); (3) detection bias (blinding of outcome assessment); (4) attrition bias (incomplete outcome data); (5) reporting bias (selective outcome reporting); and (6) other biases [40]. The specifics of the risk of bias assessment could be referenced within Additional file 1.

### GRADE assessment

We evaluated the quality of evidence for each outcome using the Grading of Recommendations, Assessment, Development and Evaluation (GRADE) framework with four possible levels: high, moderate, low, very low [41]. Five factors could decrease the quality of the evidence: risk of bias; inconsistency; indirectness; imprecision; and publication bias [41]. Detailed assessments were presented in Additional file 2. Any difference of evaluation results was discussed between the two teams of reviewers. A third reviewer participated to recheck the assessment and helped reach a consensus.

### Data analyses and synthesis

We applied the data (mean, SD) in the meta-analysis on the impacts of TBI. To mitigate inconsistencies across diverse scales and facilitate data amalgamation, we employed Standardized Mean Difference (SMD) in constructing the forest plot. *I*^*2*^ was used to evaluate the heterogeneity among the studies in quantitative statistics. When *I*^*2*^ was reported lower than 50%, a fixed-effects model was chosen in the meta-analysis [42]. We conducted sensitivity analysis to test the stability of outcomes. In order to prevent publication bias, we carried out a funnel plot test to predict the bias. In particular, according to the differences in participants, control groups, and intervention factors, we conducted a subgroup analysis to determine which components of TBI were playing key roles in mitigating burden on caregivers. This meta-analysis was done by RevMan 5.3.

## Results

### Selection of studies

**Fig. 1:**
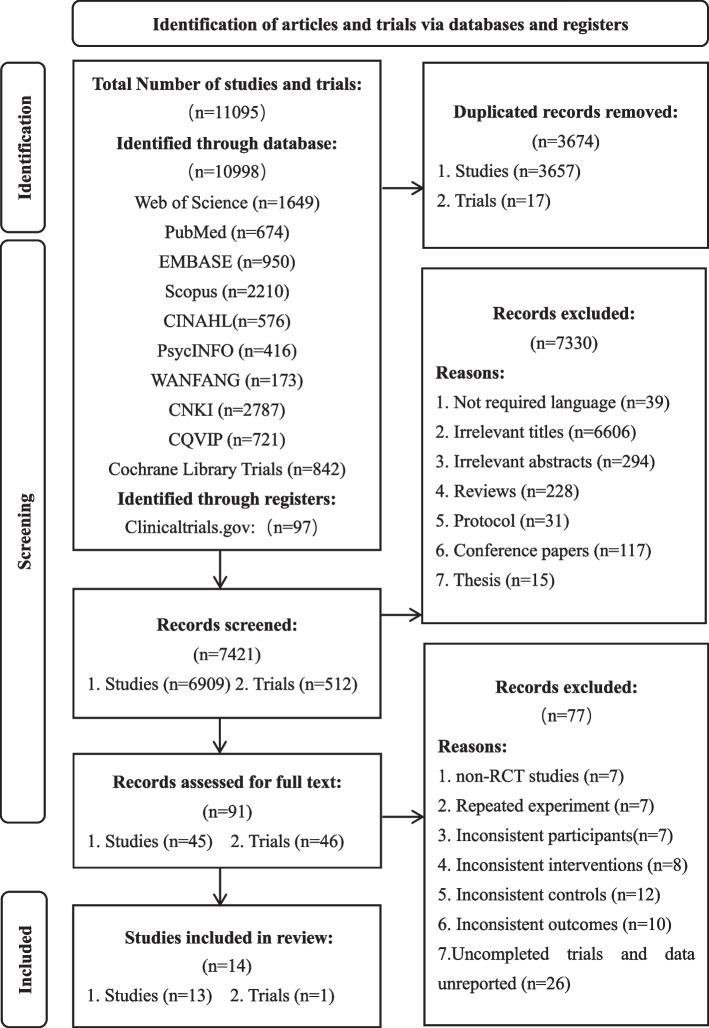
PRISMA diagram of included studies in the meta-analysis

As shown in Fig. 1, a total of 11,095 research studies and trials were identified from 11 databases and registry. After removing 3647 duplicated literature, 7421 records remained. Following the inclusion and exclusion criteria, we excluded 7330 studies as non-related, and 45 articles and 46 trials were further screened and reviewed in full text. We excluded 77 records: non-RCT studies (*n* = 7), Repeated experiment (*n* = 7), Inconsistent participants (*n* = 7), Inconsistent interventions (*n* = 8), Inconsistent controls (*n* = 12), Inconsistent outcomes (*n* = 10), Uncompleted trials and data unreported (*n* = 26). Finally, we identified 14 eligible RCTs for our meta-analysis.

### Characteristics of included studies

Across 14 trials, the sample size ranged from 10 to 237, and the total sample size was 1010. By group, 534 participants were in the experimental groups, and 476 participants were in the control groups. Caregivers’ average age was calculated as 60.65. The ages of the people under care were reported in nine studies. The mean age of care receivers ranged from 59 to 83.27 [10, 15, 16, 18–20, 22, 31, 32]. One study reported age as categorial variable and ages of all the caregivers were above 55 years [11]. Participants in the studies were all caregivers of older adults. Five studies did not report the ages of care-recipients, but all identified the care recipients as living with diseases of elderly people, namely dementia [9, 11, 12, 14, 43]. The details of the studies are presented in Table 1.

**Table 1:** Key Information about participants and RCTs in included studies

Study	Participants	Country	Medical condition	No. of participants^a^	Mean age^b^	Treatment type		Evaluation time	Measurements^c^	Results
						Control group	Experimental group			
Biliunaite et al. (2021) [22]	Caregivers	Lithuania	No medical conditions	63/54	52	Delay intervention	iCBT; Internet	8 weeks	CBI	A significantly positive change for CBI scores in the intervention group
Cristancho-Lacroix et al. (2015) [9]	Caregivers	France	Alzheimer’s disease	49/40	61.6	Usual care	CBT; Internet	3 months	ZBI	No significant differences were found on burden
De Stefano et al. (2022) [10]	family caregiver	Italy	Alzheimer’s disease	20/20	53	No intervention	Phone-based intervention	4 weeks	CBI	Significant decreased caregiver burden was revealed
Ferré-Grau et al. (2021) [31]	Nonprofessional caregivers	Spain	Chronic disease	113/92	60.65	Usual care	TIVA App	1 month	ZBI	Significant differences in ZBI scores in each group but no differences been groups
Gustafson et al. (2019) [11]	family caregivers	US	Alzheimer’s disease	25/25	-^d^	Usual care	D-CHESS; Internet	6 months	SMS	Caregiver burden worsened and all findings were nonsignificant
Hattink et al. (2015) [12]	Informal caregivers	Europe	Dementia	59/59	53.81	Delay intervention	STAR training; Internet	4 months	SMS	No effects were found on burden
James et al. (2021)	Caregivers	US	Dementia	28/10	60.3	Delay intervention	Breathing training; Internet	2 weeks	ZBI	Caregiver burden between groups did not change significantly
Kales et al. (2018) [15]	Family caregivers	US	Dementia	57/56	65.9	Delay intervention	WeCareAdvisor; Internet	1 month	ZBI	No differences between study groups on burden outcome
Levenson (2022) [43]	caregivers	US	Dementia	216/182	63.9	Delay intervention	In-Home Technology System	3 months	ZBI	-^d^
Meichsner et al. (2019) [16]	Family caregivers	Germany	Dementia	37/30	62.11	Delay intervention	Tele.TAnDemi online	8 weeks	SMS	Caregivers in the experimental and control groups did not differ in care burden
Metcalfe et al. (2019) [18]	Caregivers	England France Germany	Dementia	61/61	57.4	Delay intervention	RHAPSODY project; Internet	6 weeks	BSFC	No differences between groups were noted regarding burden
Mortenson et al. (2018) [32]	Caregivers	Canada	Disability	94/87	65.1	Usual care	Assistive technology intervention	6 weeks	CBI	The CBI did not reveal significant decreases in burden
Torkamani et al. (2014) [19]	caregivers	UK, Spain Greece	Dementia	60/58	60.69	No intervention	ALADDIN; Internet	6 months	ZBI	A significant reduction was in carer burden
Wilz et al. (2018) [20]	Family caregivers	Germany	Dementia	273/237	64.19	Usual care	Tele.TAnDem; telephone	6 months	SMS	Participants experienced a burden of care similar to that of participants at baseline tests

^a^Number of participants at baseline/participants at post intervention

^b^mean age of caregivers

^c^The Caregiver Burden Inventory (CBI), Zarit Burden Inventory (ZBI), Burden Scale for Family Caregivers (BSFC), Self-made scale (SMS)

^d^Mean age was not reported, and results were not reported in experiments’ report

### Risk of bias

**Fig. 2:**
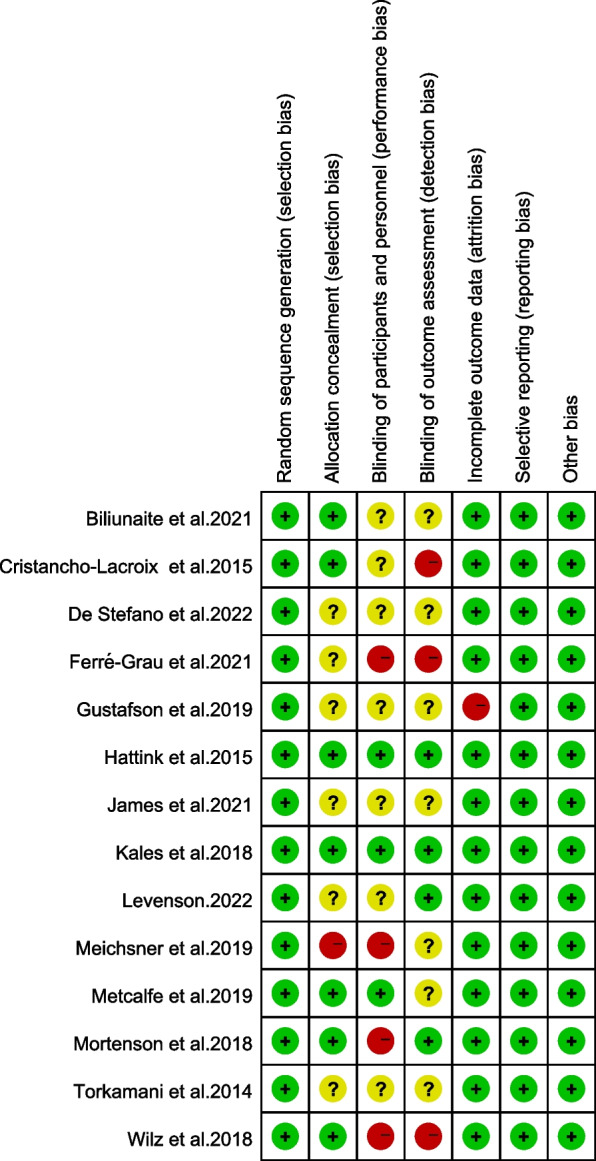
Summary of Assessments of Risk of Bias

According to Fig. 2, all studies have been rated as having a low or moderate level of risk of bias. Two studies reported a low level of risk of bias on all seven aspects [12, 15]. One study was rated with a high risk of selection bias on allocation concealment [16] and six unclear [10, 11, 14, 19, 31, 43]. Four studies were evaluated as having a high risk on performance bias [16, 20, 31, 32]. Seven trials did not report the status of blinding of participants and personnel [9–11, 14, 19, 22, 43]. Moreover, three research studies were assessed as having a high risk on detection bias [9, 20, 31]. And seven studies did not report the conditions of blinding of outcome assessment [10, 11, 14, 16, 18, 19, 22]. Except one study by Gustafson and collegues [11], the other trials studies were considered to have a low risk of bias in random sequence generation. And all trials were assessed with a low risk of bias measurement results, data reporting, and other bias.

## Results of overall effects and subgroup analyses

### Overall effects of TBI

**Fig. 3:**
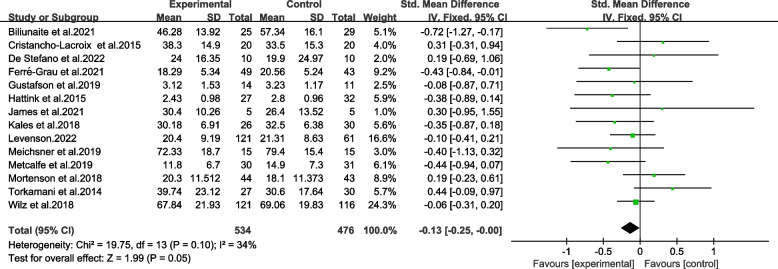
Forest plot of total effects of TBI

A meta-analysis of 14 trials indicated an overall reduction in burden of informal caregivers of older adults. As demonstrated in Fig. 3, TBI resulted in a reduction in scores of burden scale for caregivers, which dropped an average of 0.13 points (95% CI − 0.25 to − 0.00). There was statistical significance in the overall combined effects corresponding to TBI (*p* ≤ 0.05) in the sample of 1010 caregivers. It was found out that there was a low heterogeneity between the studies (Chi^2^ = 19.75, *p* > 0.05, *I*^*2*^ = 34%). The results revealed moderate quality evaluated by GRADE (Table 2).

**Table 2:** Summary of the results

Outcome or Subgroups		Number of studies	Intervention/control	Standardized mean differences (SMDs)		Heterogeneity		*p*	GRADE
				control group Mean range	experiment group SMDs (95%CI)	I^2^(%)	*p*		
Overall effect of TBI		14	534/476	2.8–79.4	− 0.13[− 0.25, − 0.00]	34%	0.10	0.05	⨁⨁⨁◯Moderate^a^
Specific medical conditions	dementia	11	416/361	2.8–79.4	−0.09[− 0.23,0.05]	8%	0.37	0.22	⨁⨁⨁◯Moderate^a^
	disability	1	44/43	18.1	0.19[−0.23,0.61]	–	–	0.38	⨁⨁◯◯Low^b^
	chronic disease	1	49/43	20.56	−0.43[− 0.84, − 0.01]	–	–	0.04	⨁⨁⨁◯Moderate^a^
	without specific medical conditions	1	25/29	57.34	−0.72[−1.27, − 0.17]	–	–	0.01	⨁⨁⨁◯Moderate^a^
Formats of technologies	ICTs	10	238/246	2.8–79.4	−0.24[− 0.42, − 0.06]	41%	0.09	0.008	⨁⨁⨁◯Moderate^a^
	telephone	2	131/126	19.9–69.06	−0.04[− 0.28,0.21]	0	0.60	0.75	⨁⨁◯◯Low^b^
	assistive technology	1	44/43	18.1	0.19[−0.23,0.61]	–	–	0.38	⨁⨁◯◯Low^b^
	smart home technology	1	121/61	21.31	−0.10[− 0.41,0.21]	–	–	0.52	⨁⨁◯◯Low^b^
Usage of TBI	psychosocial interventions	5	191/190	19.9–79.4	−0.12[− 0.32,0.08]	48	0.11	0.24	⨁⨁⨁◯moderate^a^
	problem and coping strategies	7	217/220	2.8–32.5	−0.15[− 0.34,0.04]	48	0.07	0.11	⨁⨁⨁◯moderate^a^
	behavioral training	1	5/5	26.4	0.30[− 0.95,1.55]	–	–	0.64	⨁⨁◯◯Low^b^
	home environment	1	121/61	21.31	−0.10[− 0.41,0.21]	–	–	0.52	⨁⨁◯◯Low^b^
Controlled intervention	usual care	5	248/233	3.23–69.06	− 0.05[− 0.23,0.13]	30	0.22	0.57	⨁⨁⨁◯Moderate^a^
	delay intervention	7	249/203	2.8–79.4	− 0.30[− 0.49, − 0.11]	0	0.52	0.002	⨁⨁⨁◯Moderate^a^
	No intervention	2	37/40	19.9–30.6	0.37[− 0.08,0.83]	0	0.62	0.10	⨁⨁◯◯Low^b^
Burden instruments	ZBI	6	248/189	20.56–33.5	−0.08[− 0.28,0.11]	46	0.10	0.41	⨁⨁⨁◯Moderate^a^
	CBI	3	79/82	18.1–57.34	−0.10[− 0.42,0.21]	72	0.03	0.52	⨁⨁◯◯Low^b^
	BSFC	1	30/31	14.9	−0.44[−0.94,0.07]	–	–	0.09	⨁⨁◯◯Low^b^
	Self-made scales	4	177/174	2.8–79.4	−0.14[−0.35,0.07]	0	0.63	0.19	⨁⨁⨁◯Moderate^a^
Cultural context	Europe	9	324/326	2.8–79.4	−0.17 [−0.35,0.07]	50	0.04	0.03	⨁⨁⨁◯Moderate^a^
	North America	5	210/150	3.23–32.5	−0.14[−0.32,-0.01]	0	0.58	0.63	⨁⨁⨁◯Moderate^a^
Sensitivity analysis	Exclude largest sample	13	413/360	2.8–79.4	−0.05[−0.27, 0.16]	38	0.08	0.04	⨁⨁⨁◯Moderate^a^

GRADE framework was used to evaluate the quality of evidence. High quality: where the real effect is similar to a credible estimate; Moderate quality: where the true effect is closest to the estimated effect; Low quality: where the actual effect may be significantly different from the estimated effect; and Very low quality: where the actual effect is likely to be significantly different from the estimated effect

*CI* Confidence interval

^a^The reviewers downgraded 1 point on the quality of evidence for this group because the studies reported unclear or high risk of bias in allocation concealment, blinding of participants and personnel, binding of outcome assessment, and incomplete outcomes data

^b^The reviewers downgraded 2 points on the quality of evidence for this group because the studies reported unclear or high risk of bias in allocation concealment, blinding of participants and personnel, binding of outcome assessment, and incomplete outcomes data; and with a wide confidence interval (CI)

### Results of subgroup analysis

#### Subgroup analysis of specific medical conditions

Most of the trials focused on caregivers of older adults with dementia [9–12, 14–16, 18–20, 43]. Two studies had care recipients with disability [32] and chronic diseases [31]. One study did not categorize the data on medical conditions of the care receivers [22].

**Fig. 4:**
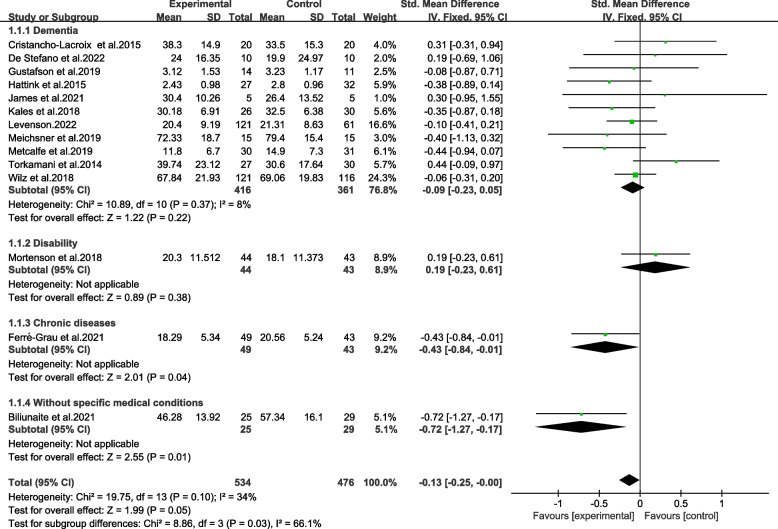
Effects among subgroups on medical conditions of older adults

And the subgroups based on care recipients’ medical conditions were significantly different (Chi^2^ = 8.86, *p* ≤ 0.05, *I*^*2*^ = 66.1%) (Fig. 4). The subgroup with older adults having chronic diseases was statistically effective (SMD = − 0.43, 95%CI − 0.84 to − 0.01, *p* < 0.05), as was the groups without specific medical conditions (SMD = − 0.72, 95%CI − 1.27 to − 0.17, *p* ≤ 0.01). Evaluated by GRADE, the results were proved with moderate and low quality (Table 2).

#### Subgroup analysis of formats of technologies

There were three kinds of technology used in intervention: (1) ICTs, including Internet-based intervention [9, 11, 12, 14–16, 18, 19, 22] and app-based intervention [31]; (2) telephone [10, 20]; (3) assistive technology [32]; and (4) smart home technology [43].

**Fig. 5:**
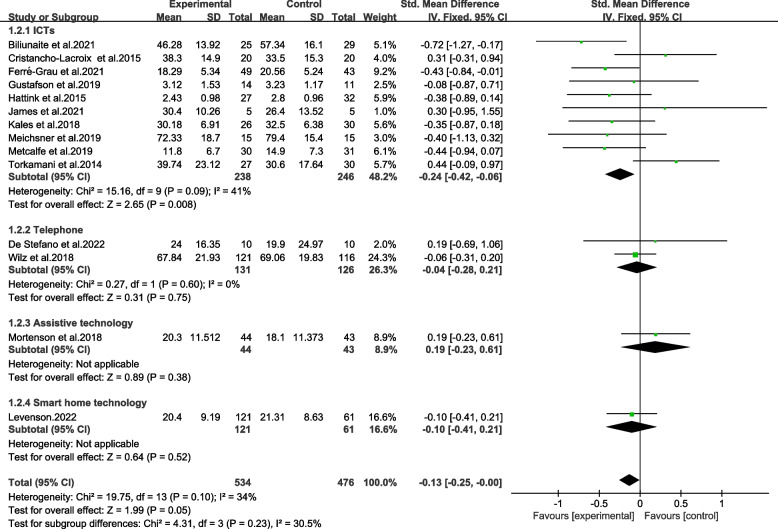
Effects among subgroups on formats of technologies

The subgroups categorized on formats of technology, including ICTs, telephone, assistive technology, and smart home technology were not significantly different (Chi^2^ = 4.31, *p* > 0.05, *I*^*2*^ = 30.5%) (Fig. 5). ICTs-based interventions had a significantly positive effect on reducing burden (SMD = -0.24, 95%CI − 0.42 to − 0.06, *p* < 0.01). According to GRADE, the results were proved in moderate and low levels of quality (Table 2).

#### Subgroup analysis of usage of TBI

Five studies offered participants psychosocial interventions [9, 10, 16, 20, 22]. Seven studies provided participants with TBI for problem and coping strategies to coordinate care [11, 12, 15, 18, 19, 31, 32]. One study offered technology-based behavioral training for caregivers of elderly people [14]. And one trial provided safe and supportive home environment [43].

**Fig. 6:**
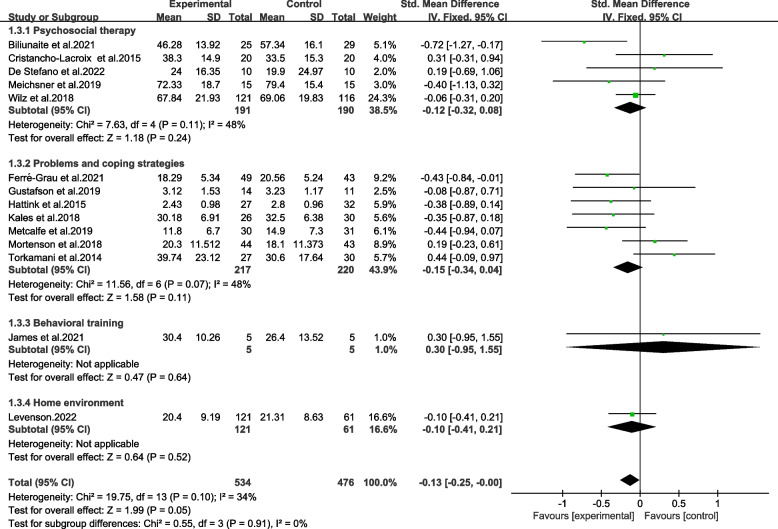
Effects among subgroups on usage of TBI

The subgroups with groups were not significantly different (Chi^2^ = 0.55, *p* > 0.05, *I*^*2*^ = 0%) (Fig. 6). Also, each group did not show significantly beneficial effect on burden (p > 0.05). The results were proved with moderate and low quality by GRADE (Table 2).

#### Subgroup analysis of controlled intervention

In the control groups, five studies provided usual care or standard care for participants [9, 11, 20, 31, 32]. The control intervention in seven trials was delay intervention [12, 14–16, 18, 22, 43]. And controlled arms did not receive any intervention in two studies [10, 19].

**Fig. 7:**
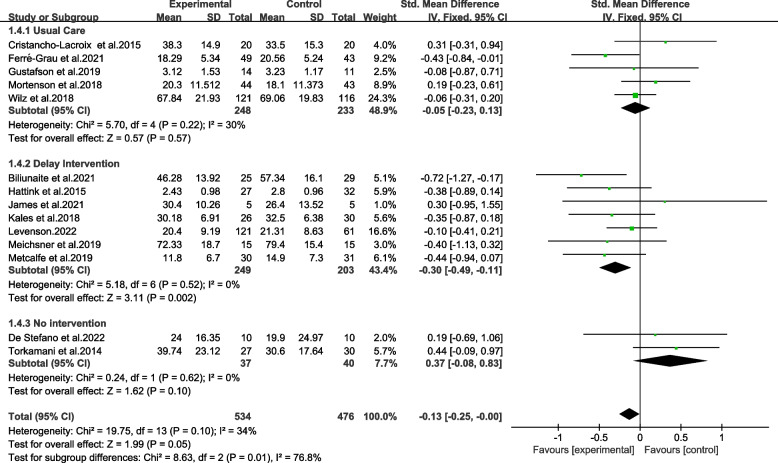
Effects among subgroups on controlled interventions

The subgroups with interventions on control group were also significantly different and highly heterogeneous (Chi^2^ = 5.86, *p* ≤ 0.01, *I*^*2*^ = 76.8%) (Fig. 7). Delay intervention as a comparator had a statistically significant effect on reducing burden on caregivers (SMD = -0.30, 95%CI − 0.49 to − 0.11, *p* < 0.01). The results were shown in moderate and low quality via GRADE (Table 2).

#### Subgroup analysis of burden instruments

Six trials measured caregivers burden levels with Zarit Burden Inventory (ZBI) [9, 14, 15, 19, 31, 43]. Three studies used The Caregiver Burden Inventory (CBI) [10, 22, 32]. One research applied Burden Scale for Family Caregivers (BSFC) [18]. And the other four studies used self-made scales (SMS) to test burden status [11, 12, 16, 20].

**Fig. 8:**
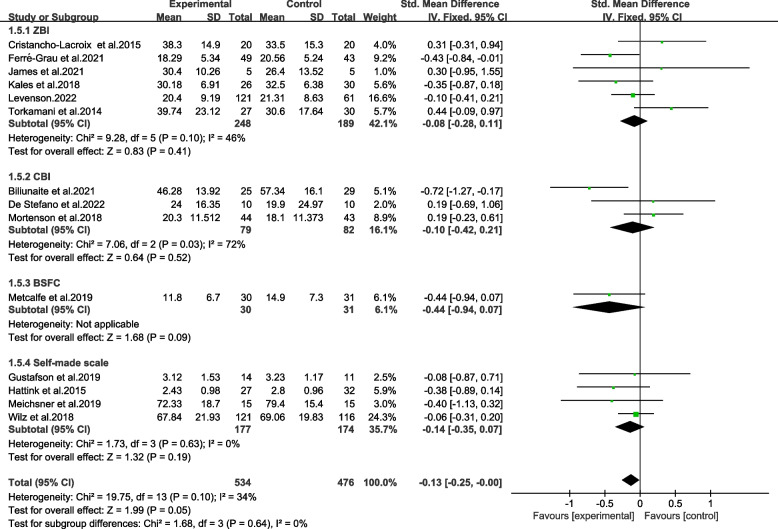
Effects among subgroups on burden instruments

The subgroups were divided by instruments of burden were not significantly different (Chi^2^ = 1.68, *p* > 0.05, *I*^*2*^ = 0%) (Fig. 8). Also, each group did not show significantly beneficial effect on burden (*p* > 0.05). Evaluated by GRADE, the results were proved in moderate and low quality (Table 2).

#### Subgroup analysis of cultural context

The studies were conducted in European and North American cultural contexts. Nine studies were set in Europe [9, 10, 12, 16, 18–20, 22, 31] and five in North American countries [11, 14, 15, 32, 43].

**Fig. 9:**
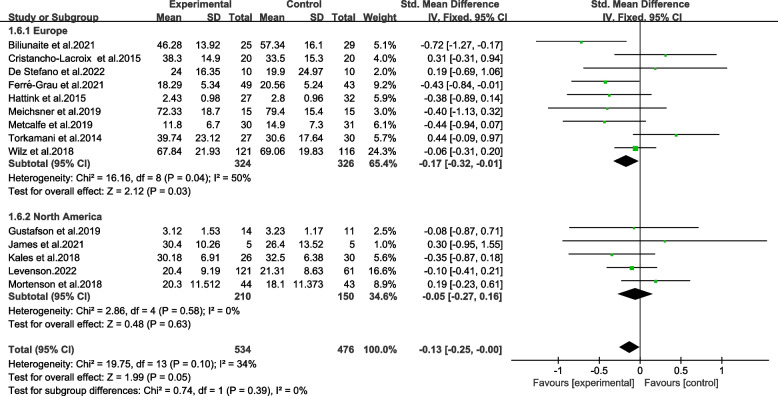
Effects among subgroups on cultural context

The subgroups categorized on cultural context were not significantly different (Chi^2^ = 0.74, p > 0.05, *I*^*2*^ = 0%) (Fig. 9). Among European context setting, the intervention had a significantly positive effect on reducing burden (SMD = -0.17, 95%CI − 0.32 to − 0.01, *p* < 0.05). The results were proved with moderate quality by GRADE (Table 2).

### Results of sensitivity analysis

**Fig. 10:**
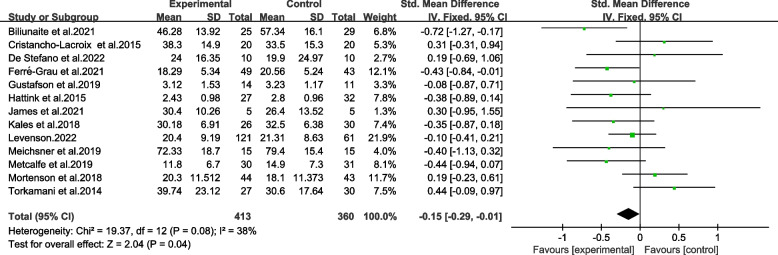
Forest plot of sensitivity analysis

To verify the stability of the meta-analysis results, we excluded a single study with the largest sample size to test its impact on the overall results. We excluded the study of Wilz et al. [20], an RCT from Germany with 121 participants in the experimental group and 116 participants in the control group. As shown in Fig. 10, the overall effect was still statistically significant (*p* < 0.05), indicating that the overall result of the combination was stable. The results also revealed moderate quality evaluated by GRADE (Table 2).

### Publication bias assessment

**Fig. 11:**
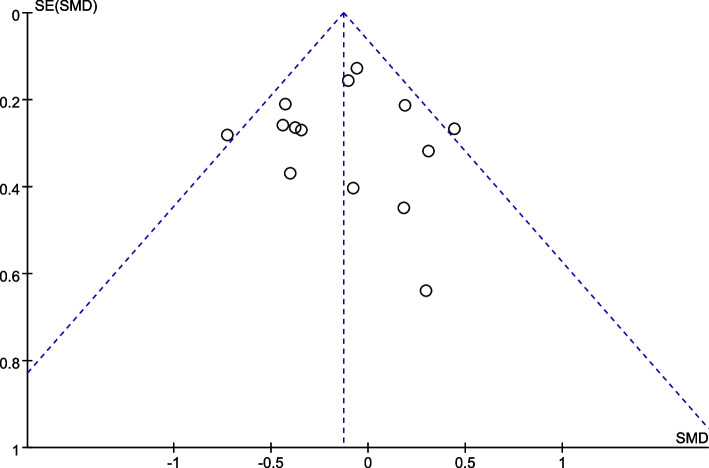
Funnel plots of effect sizes for TBI

Figure 11 depicted a funnel plot of effect sizes on the overall meta-analysis. No study fell outside the confidence interval (95%CI), indicating a low heterogeneity in this study. The plot presented symmetry, and all of the scattered points fell to the left of OR = 1, proving that there was little or no publication bias in this review.

## Discussion

Many reviews have revealed positive effects of TBI on mental health outcomes of caregivers [37]. However, this is the first systematic review and meta-analysis to include RCT researches aimed at reducing the burden on older adults’ caregivers via various technologies generally. The overall results of these studies indicated that TBI indeed ameliorated the burden on the caregivers of older adults. The results are consistent with previous studies which revealed a significant post-intervention effect of TBI on caregiver burden [2]. It could be explained technology helped maintain or improve individual capability to do things in daily life and assisted persons in coping with a range of difficulties, such as mobility [8, 17]. Caregivers often sought information and support on the web, which helped reduce the burden by caregiving [25].

Based on the findings from subgroups analysis, we’ve distinguished interventions in control groups and medical conditions of care-recipients were both determinate factors on caregivers’ burden in TBI. It was found out that the delayed intervention with TBI was significantly more beneficial to these caregivers, compared with usual care or non-intervention. It supported the opinion that TBI had the potential to reduce the burden of caregivers of older adults. As suggested previously, face-to-face delivery of interventions was not always optimal or practical for caregivers [44]. Likewise, TBI had a positive effect on caregivers of persons with chronic diseases and without specific medical conditions compared with those caregivers on persons with dementia and disability. This was in accordance with the majority of reviews that web-based intervention programs had positive effects on reducing strain on caregivers of adults with a chronic disease [25]. For utilizing TBI to reduce caregiving burden, there were no significant disparities in term of formats and usage of technology, burden instruments, and cultural context unexpectedly.

Meanwhile, TBI has revealed advantages in reducing caregiving burden of older adults with ICTs (Internet and apps) compared with telephone, assistive technology, and smart home technology. This finding was supported by an explanation that caregivers selected Internet as convenient access to individualized practical advice and emotional support to contact with professionals online, as well as helpful information about the disease, coping skills, and other information to support caregiving [36]. Apps became indispensable and complementary facilitators to health care [23]. Moreover, different from trials in North American countries, TBI conducted in European countries displayed significant effects. It might be explained technologies had more advantages in application within European humanistic or geographic conditions. Similarly, in this review BSFC was proved as an instrument with advantages in measuring caregiving burden of informal caregivers, which was might for the reason on the construction of the instrument. However, usage of technology in intervention consisted of psychosocial intervention, problems and coping strategies, behavioral training, and home environment did not show any disparities and advantages in TBI. On this sense, we did not have preferable contents in using technology to reduce caregivers’ burden.

### Strengths and limitations

This review has several strengths. It includes the most recent evidences in exploring the potential effects of TBI on the burden of caregivers of older adults. All the selected trials have been published since 2014. And it ensures literature diversity and comprehensiveness for including peer review articles, grey literature, and registry of RCTs. Conclusively, it proves the positive effects on caregiving burden of older adults’ carers via TBI generally. Via subgroup analyses, this review has also distinguished the most effective factors and advantages in exploring the mechanism of TBI to work on caregiver burden. Furthermore, sensitivity analysis reveals the results of meta-analysis are stable, and the results of publication bias test suggest less publication bias. Also, this review searches both Chinese and English studies to avoid bias in cultural diversity.

However, there remain some limitations. Even included RCTs are mostly conducted with rigorous experimental designs, several of the items are evaluated with high or unclear risk. Evaluations by GRADE assert moderate quality of overall effects and sensitivity analysis, and moderate and low quality on results of subgroup analysis, which are due to risk of bias and imprecision. In one study, the final standard deviation is unavailable, and it was be substituted by the baseline SD following Cochrane Systematic Review Handbook [11]. Also, one trial was excluded for lack of key data. And a number of the trial protocols and reports have not been completed or published.

### Implications

Technology for caregivers of older adults is in a rapid pace of changes and developments. It is a policy issue for both of the public and private sectors tend to utilize technologies to facilitate aspects of caregiving to address caregiving needs. The developments of TBI for caregiving demands urgent policy advocacy. Government or organizations like nursing associations are expected to make standards, including notions, categories, and agendas on technologies for caregiving. Nursing professionals should participate in cross-disciplinary dialogues on caregiving-related models and measurements which could be processed creatively in technology for caregiving. It is promising to apply technologies in reducing burden and supporting informal caregivers of aging persons. Yet, there were not that much studies on practice of TBI to help caregivers. It still needs to raise awareness and perceptions about utilization of technologies for caregiving on older caregivers and could refer to European experience. Clinical professionals should pay attention on the factors of caregivers’ adoption of technology, containing social/demographic factors (e.g., socio-economic status), attitudinal elements (e.g., computer anxiety), and component abilities (e.g., cognitive abilities) [24, 27, 33], in order to make caregivers feel more efficacy to use TBI as much as possible. Meanwhile, most of published researches are about TBI in dementia caregiving and by means of ICTs. We advocate its application in caregiving on older adults with various medical conditions, and more kind of modalities of technologies could be tried to support them. It has been proved tailored systems gained more chance of acceptance by the target population [6]. And TBI should be matched to caregivers’ needs seamlessly [21]. As discussed previously, rigorous evaluations were lacking to evaluate the effects of TBI [30]. Yet, as referred in included studies, it is impossible to blind participants when they are exposed online during intervention [16, 20]. We should consider preventing the risks in RCT research designs for TBI. Also, additional methodologies, such as Egger regression, could be implemented to enhance result reliability and precision in accordance with the demands of future investigations. Further studies are warranted to elucidate the associations between intervention effect sizes and various characteristics of RCTs, thus elucidating the efficacious mechanisms of TBI more comprehensively. Furthermore, while insights from European and North American studies are valuable, caution is advocated when applying these findings directly to the Asian region. We suggest more researches with high-quality on TBI to reduce the burden of caregivers published in Eastern contexts.

## Conclusion

Highly reliable evidence about applying TBI for reducing caregiving burden remains limited. This research is the first review to discuss on the benefits of a variety of technological interventions on the burden of caregivers, devoted to the caregiving of older adults with dementia, disability, chronic diseases, and without medical conditions. TBI has been elaborated as a positive intervention to alleviate the burden on these caregivers. Controlled intervention and medical characteristics of care recipients are both influencing factors on effects of TBI to reduce burden of caregivers, among which we could focus more on caregivers of persons with chronic diseases and without medical conditions, and delayed TBI as controls. It also enlightens us to pay more attention on the advantages of ICTs-based interventions, BSFC as a burden measurement, and the application of TBI in European context. Moreover, more RCTs with high-quality, different groups of participants, modalities of TBI, and caregiver outcomes are expected in future studies, in order to enrich the evidence of reduction of burden by TBI for caregivers of older adults.

### Supplementary Information

Supplementary Material 1.

Supplementary Material 2.

## Data Availability

The authors confirm that the data supporting the findings of this study are available within the article and its supplementary materials.

## Abbreviations

TBI: Technology-based interventions
RCTs: Randomized controlled trials studies
CI: Confidence interval
PRISMA: Preferred Reporting Items for Systematic Review and Meta-analyses
GRADE: The Grading of Recommendations, Assessment, Development and Evaluation
CBI: The Caregiver Burden Inventory
ZBI: Zarit Burden Inventory
BSFC: Burden Scale for Family Caregivers
SMS: Self-made scale
