# Supplementary material for: Technology-based interventions on burden of older adults’ informal caregivers: a systematic review and meta-analysis of randomized controlled trials
Source: BMC Geriatr. 2024 May 4;24:398. doi: 10.1186/s12877-024-05018-w (PMC11070124; doi:10.1186/s12877-024-05018-w)
Supplement: Supplementary file 2 — Supplementary Material 2. [file 12877_2024_5018_MOESM2_ESM.docx]

GRADE assessment results

|  | | risk of bias | inconsistency | indirectness | imprecision | publication bias | score | conclusion |
| --- | --- | --- | --- | --- | --- | --- | --- | --- |
| Overall effect of TBI | | -1 | 0 | 0 | 0 | 0 | -1 | ⨁⨁⨁◯Moderate^1^ |
| 3.4.2 Care-recipients with specific medical conditions | dementia | -1 | 0 | 0 | 0 | 0 | -1 | ⨁⨁⨁◯Moderate^2^ |
|  | disability | -1 | 0 | 0 | -1 | 0 | -2 | ⨁⨁◯◯Low^3^ |
|  | chronic disease | -1 | 0 | 0 | 0 | 0 | -1 | ⨁⨁⨁◯Moderate^4^ |
|  | without specific medical conditions | -1 | 0 | 0 | 0 | 0 | -1 | ⨁⨁⨁◯Moderate^5^ |
| 3.4.3 formats of technologies | ICTs | -1 | 0 | 0 | 0 | 0 | -1 | ⨁⨁⨁◯Moderate^6^ |
|  | telephone | -1 | 0 | 0 | -1 | 0 | -2 | ⨁⨁◯◯Low^7^ |
|  | assistive technology | -1 | 0 | 0 | -1 | 0 | -2 | ⨁⨁◯◯Low^3^ |
|  | smart home technology | -1 | 0 | 0 | -1 | 0 | -2 | ⨁⨁◯◯Low^8^ |
| 3.4.4 usage of TBI | psychosocial interventions | -1 | 0 | 0 | 0 | 0 | -1 | ⨁⨁⨁◯moderate^7^ |
|  | problem and coping strategies | -1 | 0 | 0 | 0 | 0 | -1 | ⨁⨁⨁◯moderate ^9^ |
|  | behavioral training | -1 | 0 | 0 | -1 | 0 | -2 | ⨁⨁◯◯Low^10^ |
|  | home environment | -1 | 0 | 0 | -1 | 0 | -2 | ⨁⨁◯◯Low^8^ |
| 3.4.5 controlled intervention | usual care | -1 | 0 | 0 | 0 | 0 | -2 | ⨁⨁⨁◯Moderate ^7^ |
|  | delay intervention | -1 | 0 | 0 | 0 | 0 | -1 | ⨁⨁⨁◯Moderate^11^ |
|  | No intervention | -1 | 0 | 0 | -1 | 0 | -2 | ⨁⨁◯◯Low^12^ |
| 3.4.6 burden instruments | ZBI | -1 | 0 | 0 | 0 | 0 | -2 | ⨁⨁⨁◯Moderate ^9^ |
|  | CBI | -1 | 0 | 0 | -1 | 0 | -2 | ⨁⨁◯◯Low^13^ |
|  | BSFC | -1 | 0 | 0 | -1 | 0 | -2 | ⨁⨁◯◯Low^14^ |
|  | self-made scales | -1 | 0 | 0 | 0 | 0 | -2 | ⨁⨁⨁◯Moderate ^15^ |
| 3.4.7 Cultural context | Europe | -1 | 0 | 0 | 0 | 0 | -1 | ⨁⨁⨁◯Moderate^16^ |
|  | North America | 0 | 0 | 0 | 0 | 0 | -1 | ⨁⨁⨁◯Moderate^17^ |
| Sensitivity | Exclude largest sample | -1 | 0 | 0 | 0 | 0 | -1 | ⨁⨁⨁◯Moderate^1^ |

^1^The reviewers downgraded 1 point on the quality of evidence for overall effects because 12 trials reported unclear or high risk of bias in allocation concealment, blinding of participants and personnel, binding of outcome assessment, and incomplete outcomes data.

2 The reviewers downgraded 1 point on the quality of evidence for this group because 9 trials reported unclear or high risk of bias in allocation concealment, and blinding of participants and personnel.

3 The reviewers downgraded 2 points on the quality of evidence for this group because the study reported high risk of bias in incomplete outcomes data; and with a wide confidence interval (CI).

4 The reviewers downgraded 1 point on the quality of evidence for this group because the study reported unclear or high risk of bias in allocation concealment, blinding of participants and personnel, and binding of outcome assessment

5 The reviewers downgraded 1 point on the quality of evidence for this group because the study reported unclear risk of bias in blinding of participants and personnel and binding of outcome assessment

6 The reviewers downgraded 1 point on the quality of evidence for overall effects because 8 trials reported unclear or high risk of bias in allocation concealment, blinding of participants and personnel, and binding of outcome assessment, and incomplete outcomes data.

7 The reviewers downgraded 2 points on the quality of evidence for this group because the studies reported unclear or high risk of bias in allocation concealment, blinding of participants and personnel, binding of outcome assessment, and incomplete outcomes data; and with a wide confidence interval (CI).

8 The reviewers downgraded 1 point on the quality of evidence for this group because the study reported unclear risk of bias in allocation concealment and blinding of participants and personnel; and with a wide confidence interval (CI).

9 The reviewers downgraded 2 points on the quality of evidence for this group because 5 studies reported unclear or high risk of bias in allocation concealment, blinding of participants and personnel, binding of outcome assessment, and incomplete outcomes data; and with a wide confidence interval (CI).

10 The reviewers downgraded 1 point on the quality of evidence for this group because the study reported unclear risk of bias in allocation concealment, blinding of participants and personnel, and binding of outcome assessment; and with a wide confidence interval (CI).

11The reviewers downgraded 1 point on the quality of evidence for this group because 5 studies reported unclear or high risk of bias in allocation concealment, blinding of participants and personnel, binding of outcome assessment, and incomplete outcomes data

12 The reviewers downgraded 2 points on the quality of evidence for this group because the studies reported unclear risk of bias in allocation concealment, blinding of participants and personnel, binding of outcome assessment, and incomplete outcomes data; and with a wide confidence interval (CI).

13 The reviewers downgraded 2 points on the quality of evidence for this group because the studies reported unclear or high risk of bias in allocation concealment, blinding of participants and personnel, and binding of outcome assessment; and with a wide confidence interval (CI).

14 The reviewers downgraded 1 point on the quality of evidence for this group because the study reported unclear risk of bias in binding of outcome assessment; and with a wide confidence interval (CI).

15 The reviewers downgraded 2 points on the quality of evidence for this group because 3 studies reported unclear or high risk of bias in allocation concealment, blinding of participants and personnel, binding of outcome assessment, and incomplete outcomes data; and with a wide confidence interval (CI).

16 The reviewers downgraded 1 point on the quality of evidence for this group because 8 studies reported unclear or high risk of bias in allocation concealment, blinding of participants and personnel, and binding of outcome assessment

17 The reviewers downgraded 1 point on the quality of evidence for this group because 4 studies reported unclear or high risk of bias in allocation concealment, blinding of participants and personnel, binding of outcome assessment, and incomplete outcomes data.
